# Editorial: The mechanism of immune cells in the development of inflammatory bowel disease (IBD) and colitis-associated colorectal cancer (CAC)

**DOI:** 10.3389/fimmu.2023.1218958

**Published:** 2023-05-19

**Authors:** Edda Russo, Kai Yin, Xiumei Sheng, Fei Mao, Amedeo Amedei

**Affiliations:** ^1^ Department of Experimental and Clinical Medicine, University of Florence, Florence, Italy; ^2^ Department of General Surgery, Affiliated Hospital of Jiangsu University, Institute of Digestive Diseases, Jiangsu University, Zhenjiang, China; ^3^ Institute of Digestive Diseases, Jiangsu University, Zhenjiang, Jiangsu, China

**Keywords:** immunology & inflammation, inflammatory bowel disease, colitis associated cancer (CAC), immune cell, immunotherapy

Inflammatory bowel disease (IBD), which includes Crohn’s disease (CD) and ulcerative colitis (UC), is characterized by intermittent chronic inflammation of the gastrointestinal tract, due to a dysregulated immune response against intestinal mucosa, leading to bowel damage (1, Russo et al.). In IBD, the sealed intercellular junctions are damaged, either from a primary barrier function defect or from severe inflammation. Excessive inflammatory response leads to continued deterioration of the epithelium and exposure to the intestinal microbiome (bacteria, fungi, viruses, and their genes living in the gut), thereby further worsening the inflammation (2, 3).

Colitis-associated colorectal cancer (CAC) is the most critical IBD complication (Rubin et al.). The carcinogenesis in IBD is a multistep progression, due to increasing degrees of histologic, molecular, and endoscopic alteration, from no dysplasia against chronic inflammation to low-grade dysplasia to high-grade dysplasia and eventually neoplasia (4).

In addition, inflammation and cancer are closely related, as it has been well-documented (5, 6). It induces genetic and epigenetic alterations transmissible to epithelial cell progeny (7), promoting epithelial turnover. Finally, inflammation promotes microbiome dysbiosis, contributing to CAC through the production of carcinogens as well as reactive metabolites, disrupting the epithelial barrier (8). As a result, an increased intestinal permeability induces a persistent stimulation of the mucosal immune response, leading to chronic inflammation (8).

The intestinal microenvironment, comprised of three elements (epithelium, local immune system, and intestinal microbiome), plays a crucial role in IBD development and in the process leading to CAC (9). Each of these elements is interconnected and crucial to maintaining gut homeostasis. However, the immune cells, including monocytes, macrophages, myeloid-derived suppressor cells [MDSCs], neutrophils, and dendritic cells [DCs], are the main component of the local microenvironment, exerting specific functions in intestinal tissue during IBD and CAC. In fact, they can: i. produce inflammatory cytokines, exosomes, and other cell factors; ii. modulate the functions of other cells, like fibroblasts, in cell contact-dependent ways; and iii. have an immunomodulatory role for intestinal epithelial cells, which is critical to maintaining the gut immune homeostasis in tumours (9).

In this landscape, this Research Topic provides an overview of immune cell roles in modulating intestinal epithelial cell functions in colon tissue immunopathology, such as inflammation, IBD and CAC, welcoming all those studies, performed in human IBD/CAC and animal models of colitis, which will help to elucidate cellular and molecular mechanisms driving tissue immuno-pathogenesis. In addition, the Research Topic examines the role of the immune microenvironment in the development of IBD and CAC by modulating inflammatory factors and the intestinal microbiome.

In this regard, the manuscript of Zhou et al. examines the mechanisms of inflammatory mediators in the IBD and colorectal cancer (CRC) microenvironment. It addresses the role of lactate, an intermediate by-product of glucose metabolism found in the gut microenvironment, in the inflammatory process. The authors examined its inflammatory/anti-inflammatory effect with a double approach: an *in vitro* study on macrophages stimulated by LPS and, for the first time, an “*in vivo”* study on a mouse model of DSS-induced colitis. They observed an inhibitory effect of lactate in the TLR/NF-κB signaling pathway and a promotion of the polarization of macrophages in the colonic tissue, inducing pro-inflammatory factors. In addition, the authors reported lactate involvement in the mucosal barrier-repairing process and in the protection of the intestinal tissue in the inflammatory condition. Therefore, they finally propose lactate as a promising and effective drug for treating inflammation through immunometabolism regulation.

As previously reported, CAC is considered the most serious complication of IBD; however, there is still a lack of clear understanding of its pathogenesis from UC (Rubin et al.). In this context, the study of Zhang et al. improved the understanding of the role of neutrophils in UC and in its progression to CAC, providing new and more effective insights into CAC prevention and treatment. With the aim to identify differentially expressed genes and potential biological pathways, the authors first used a bioinformatic approach to analyse UC transcriptomic data. Using validation cohorts and mouse models, they observed an increased infiltration of neutrophils in UC tissue and augmented MPO and pSTAT3 expression. Thus, they classified the UC patients into two subtypes of neutrophil infiltration; interestingly, highly infiltrate of subtype B neutrophils of UC patients showed a higher risk of developing CAC. The authors suggested that neutrophils might promote the conversion of UC into CAC.

On the other hand, the mechanisms regulating the dysfunctional immune cells in the intestine and inflammatory phenotypes in UC are still unclear. In the study of Pan et al. a differential expression analysis on microarray datasets, as well as a pathway enrichment analyses, were conducted to identify common differentially expressed genes in UC patients. Further algorithms were used to determine the infiltration status of immune cells and the correlation with hub genes. The authors demonstrated the involvement of CCL3, MMP3, and TIMP1 genes in the UC pathogenesis and activity. Indeed, active UC patients who responded to Golimumab therapy exhibited a decrease in their expression in the gut mucosa. The expression of these three genes was positively correlated with dendritic cells, macrophages, CD8^+^ T cells, and neutrophils in CRC patients. So, the authors suggest these three genes as novel pharmacological regulators of UC and as biomarkers for immune cell infiltration in CRC.

Another relevant issue addressed in this Research Topic concerns the molecular mechanisms underlying the complex interaction between immune cells and intestinal epithelial cells in CAC immune surveillance and tumor evasion, which have not been currently identified. Currently, the advent of immunotherapy is changing the way we think about cancer treatment (10). Immunotherapy includes immune checkpoint inhibitors (ICIs), adoptive cell therapy (ACT), cancer vaccines and cytokines, aiming to improve the ability of the immune system to recognize, target and eliminate cancer cells. ICI-based immunotherapy has shown effective clinical outcomes in immunologically “hot” tumors. However, for immunologically “cold” tumors such as CRC, only a small number of patients are currently benefiting from ICIs, due to individual differences and low response rates. Yuan et al. summarized the role of immunotherapy in CRC in a well-articulated review. The authors also propose a breakthrough and strategy to improve the role of immunotherapy in cold CRC based on its characteristics.

Moreover, technological advances and the introduction of novel therapeutic approaches (11–13), in clinics have led to unique insights into the regulation of aberrant immune responses in IBD. In this perspective, the single-cell RNA-sequencing (scRNA-seq) technology has become a key technique for interrogating the transcriptome at the single-cell level and for resolving the heterogeneity of various immune subsets involved in inflammatory networks in chronic inflammation (14). To improve the interpretation of these “Big Data”, many tools such as “Scanpy”, have been developed over the past years to support scRNA-seq analyses (15–17), including integrated unsupervised clustering algorithms. However, in the case of the manual selection of specific cell subsets, these professional pipelines are complex, and tools for the manual selection and further downstream analysis of single-cell populations are missing so far. In this scenario, Dedden et al. developed scSELpy (single cell SELection python), offering to Scanpy users the ability to annotate their cells of interest by means of manual selection, This tool is useful for the selection and sub-phenotyping of T-cell subsets implicated in IBD beyond standard clustering. The authors demonstrated that scSELpy might therefore become an important application for future single-cell transcriptomic analyses, supporting future immunological research.

## Author contributions

ER wrote the manuscript, ER edited the manuscript, AA corrected the final version. All authors read and approved the manuscript.
